# Optogenetic Inhibition of Striatal Neuronal Activity Improves the Survival of Transplanted Neural Stem Cells and Neurological Outcomes after Ischemic Stroke in Mice

**DOI:** 10.1155/2017/4364302

**Published:** 2017-09-14

**Authors:** Yifan Lu, Lu Jiang, Wanlu Li, Meijie Qu, Yaying Song, Xiaosong He, Zhijun Zhang, Guo-Yuan Yang, Yongting Wang

**Affiliations:** ^1^Neuroscience and Neuroengineering Research Center, Med-X Research Institute and School of Biomedical Engineering, Shanghai Jiao Tong University, Shanghai, China; ^2^Department of Neurology, Ruijin Hospital, School of Medicine, Shanghai Jiao Tong University, Shanghai 200030, China; ^3^Department of Human Anatomy, Guangzhou Medical University, Guangzhou 510182, China; ^4^Brain Science and Technology Research Center, Shanghai Jiao Tong University, Shanghai, China

## Abstract

Neural stem cell (NSC) transplantation is a promising treatment to improve the recovery after brain ischemia. However, how the survival, proliferation, migration, and differentiation of implanted NSC are influenced by endogenous neuronal activity remains unclear. In this work, we used optogenetic techniques to control the activity of striatal neurons and investigated how their activity affected the survival and migration of transplanted NSCs and overall neurological outcome after ischemic stroke. NSCs cultured from transgenic mice expressing fluorescent protein were transplanted into the peri-infarct region of the striatum after transient middle cerebral artery occlusion (tMCAO) surgery. The striatal neurons were excited or inhibited for 15 minutes daily via implanted optical fiber after tMCAO. The results revealed that mice which received NSC transplantation and optogenetic inhibition had smaller brain infarct volume and increased NSC migration compared to the NSC alone or PBS group (*p* < 0.05). In contrast, mice which received NSC transplantation and optogenetic excitation showed no difference in infarct volume and neurological behavior improvement compared to the PBS control group. In vitro experiments further revealed that the conditioned media from excited GABAergic neurons reduced NSC viability through paracrine mechanisms. *Conclusion*. Optogenetic inhibition of striatal neuronal activity further improved neurological recovery after NSC transplantation at the subacute phase after brain ischemia.

## 1. Introduction

Ischemic stroke is a cerebrovascular disease which can result in motor, sensory, cognitive deficits and even death. Developing effective treatment and rehabilitation strategies for ischemic stroke remains a challenging task at present [1]. In recent years, studies on animal models showed that neural stem cell (NSC) transplantation holds promise for the treatment of stroke [2–5]. In the early phase after brain ischemia, transplanted NSCs can activate the endogenous repair pathways that are involved in immunomodulation [6, 7], angiogenesis [7–9], neurogenesis [9, 10], and neural plasticity [11, 12], mostly through paracrine mechanisms. In the chronic phase of stroke, transplanted NSCs can replace the lost neurons and integrate into the host circuitry by physically connecting with surrounding host cells, which may contribute to long-term recovery [13–15]. However, the therapeutic efficacy of stem cells is limited by the poor survival of transplanted cells [16]. The previous studies had unveiled the influence of various extrinsic survival factors including growth factors, morphogens, proteoglycans, cytokines, and hormones in the poststroke niche [17] on the survival and proliferation of embryonic and adult NSCs. Adult NSCs also were shown to respond to a variety of neurotransmitters such as glutamate, dopamine, histamine, GABA, D-Serine, NO, and 5-HT [17]. Action potential can lead to the secretion of neurotransmitters, suggesting that the activity of surrounding intrinsic neurons would influence the survival, proliferation, and differentiation of the transplanted NSCs. However, very limited data is available regarding how intrinsic neuronal activities influence the behavior and fate of transplanted NSCs under pathological conditions after cerebral ischemia.

In this study, we used optogenetic technique to regulate striatal neuronal activity after transient middle cerebral artery occlusion (tMCAO) followed by NSC transplantation in mice to investigate whether exciting or inhibiting striatal neuronal activity in the peri-infarct region could improve the survival and migration of transplanted NSCs.

## 2. Methods and Materials

### 2.1. Animal Experimental Design

**Figure 1:**
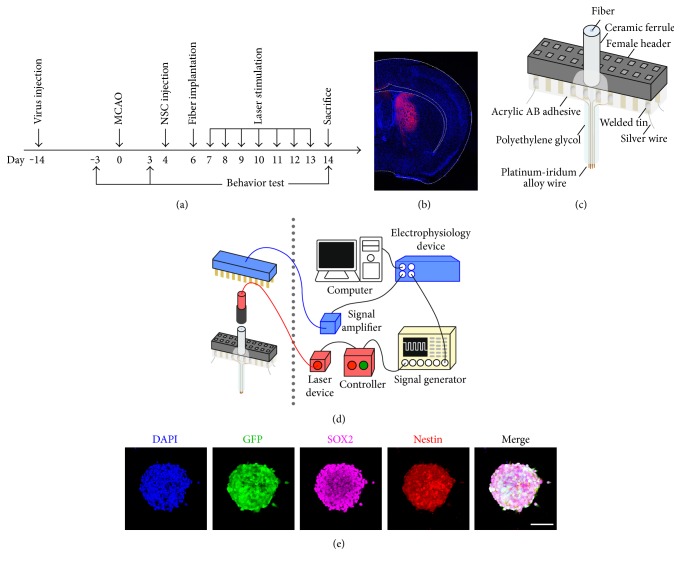
Experimental design and technical set-up. (a) The schedule of animal experiments in this study. (b) The representative expression of ChR2-mCherry opsins in striatal neurons under CaMKII promoter. (c) The schematic representation of the structure of lab-made optrode. (d) The hardware system for synchronous laser stimulation and electrophysiological recording. (e) The characterization of cultured NSC-GFP sphere by immunohistochemical staining with NSC markers SOX2 and Nestin. Bar = 100 *μ*m.

Animal experimental procedures were approved by the Institutional Animal Care and Use Committee of Shanghai Jiao Tong University, Shanghai, China. Adult male ICR mice were housed in specific-pathogen-free rooms which were maintained on a 12-hour light/dark cycle at room temperature around 25°C. Intraperitoneal ketamine/xylazine (100 mg/10 mg per kg, Sigma, San Louis, MO) was used for anesthesia. The animal experimental design for this study is shown in Figure 1(a). Sixty-four mice received virus injection, thirty-two mice were used for excitatory stimulation experiments, and the others were used for inhibitory stimulation experiments. In the excitatory or inhibitory stimulation experiments, animals were randomly divided into four groups that designed to receive (1) PBS (PBS group); (2) PBS followed by laser stimulation (PBS-E for excitation or PBS-I for inhibition groups, resp.); (3) NSC transplantation (NSC group); and (4) NSC transplantation followed by laser stimulation (NSC-E or NSC-I group, resp.) after 60-minute tMCAO surgery. PBS or NSC was administrated at 4 days after tMCAO surgery. Optical fiber was implanted at day 6. Laser stimulation was carried out from day 7 to day 13, for 15 minutes daily. Behavioral test was performed before tMCAO and at 3 and 14 days after tMCAO. Animals were sacrificed at day 14.

### 2.2. Virus Transfection

After mice were anesthetized, 0.5 microliter adeno-associated virus (AAV) (Obio technology, Shanghai) carrying ChR2-mCherry or ArchT-eGFP gene under the control of CaMKII promoter was injected into the left striatum (AP = 0 mm, ML = −2.5 mm, DV = 3 mm relative to the bregma) using a minipump (WPI, Sarasota, FL) at a rate of 50 nl/min. The titer of AAV used in this study was 4 × 10^12^ TU/ml. After finishing injection, the needle was maintained in the brain for additional 5 minutes before it was withdrawn.

### 2.3. Transient Middle Cerebral Artery Occlusion Surgery

Two weeks after virus injection, mice underwent a 60-minute tMCAO surgery. The protocol of tMCAO surgery was described in the previous study [18]. Briefly, the middle cerebral artery (MCA) was occluded by a 6–0 suture (Covidien, Mansfield, MA) with a silicone-coated round tip. During the surgery, mice body temperature was maintained at 37 ± 0.5°C using a heating pad (RWD Life Science) with a feedback control. The blood flow in MCA was measured by Laser Doppler monitor (Moor instruments, Devon, U.K.) before MCAO, at the beginning of occlusion and 1 minute after reperfusion. Animals whose MCA blood flow did not decrease to 10%~20% of baseline during occlusion or did not recover to 40%~60% of baseline after reperfusion were excluded from this study.

### 2.4. NSC Culture, Characterization, and Transplantation

NSCs were isolated from the cortex of E14 transgenic mice (Animal Research Center of Nanjing University, Nanjing, China) that express green fluorescent protein (GFP) under the control of beta-actin promoter or red fluorescent protein (RFP) under the control of Tie2 promoter, as previously described [19] and maintained by DMEM/F-12 medium (Gibco, Carlsbad, CA, USA) with B27 supplement (Gibco) and growth factors (20 ng/ml epidermal growth factor, 20 ng/ml fibroblast growth factor-2) (Gibco) at 37°C under 5% CO_2_ atmosphere. Culture medium was replaced with fresh medium every 2 days. NSC spheres were passaged at one-week intervals. The NSCs used for experiments were obtained from cultures that underwent 3–5 passages. To characterize the cultured NSCs, the cells were grown on poly-L-ornithine hydrobromide (Sigma, St. Louis, MO) and laminin (Sigma)-coated glass coverslips and immunostained with Nestin (Millipore, Billerica, MA, USA) and SOX2 (Abcam, Cambridge, MA).

NSC transplantation was performed at 4 days after tMCAO. Before transplantation, NSC spheres were incubated with accutase (Gibco) for 20 minutes to disperse into single NSCs. A total of 3 × 10^5^ NSCs dissolved in 10 *μ*l PBS was injected into the left striatum (AP = 0 mm, ML = −2.5 mm, DV = 3 mm relative to the bregma) using a minipump at 1 *μ*l/min. After finishing the injection, the needle was maintained in the brain for additional 5 minutes before it was withdrawn.

### 2.5. Optrode Preparation and *In Vivo* Electrophysiology

Optrode preparation and implantation were performed as previously described [20]. The structure of the homemade optrode was shown in Figure 1(c). The optrode was comprised of an optical fiber, with 200 *μ*m diameter and 0.22 numerical aperture (Thorlabs, NEWTON, NJ), surrounded by 8 platinum-iridium alloy microelectrodes with 35 *μ*m diameter (Plexon, Dallas, Texas). The optrode was implanted into the left striatum of the mouse (AP = −0.02 mm, ML = −2.5 mm, DV = 2 mm relative to bregma) at 3 days after tMCAO for *in vivo* electrophysiology recording. Optrode implantation and *in vivo* electrophysiology were performed on 3 mice in each group that express ChR2 or ArchT in the striatum, respectively. Electrophysiological data was synchronously recorded by electrophysiological instrument (Plexon, Dallas, Texas) when the striatal neurons were stimulated by a laser (Figure 1(d)). Single and multiunit activity recordings were sampled at 30 kHz and bandpass filtered at 250 Hz to 3000 Hz. Different laser power conditions ranged from 0.001 mW to 0.1 mW for 473 nm laser pulse in the ChR2 group and from 0.1 mW to 2 mW for 530 nm constant laser in the ArchT group were tested. At the beginning of each recording, five-minute baseline was recorded to ensure stable electrophysiological signal. All recordings were carried out with the animals under anesthesia.

### 2.6. Optical Fiber Implantation and Laser Stimulation

All animals except the 6 mice for in vivo electrophysiology underwent optical fiber implantation at 6 days after tMCAO, following the protocol as described in the previous study [20]. Laser stimulation was performed once daily from 7 to 13 days after tMCAO. Each stimulation session lasted for 15 minutes. The parameters of lasers were controlled by a waveform generator (Tektronix, Shanghai, China). For the ChR2 group, each 5-second stimulation cycle was composed of 1-second stimulation phase and 4-second rest phase. In the stimulating phase, 473 nm laser pulses with 5 ms pulse width were administrated at 20 Hz. For the ArchT group, 530 nm laser was administrated constantly for 15 minutes. The laser powers were 0.05 mW for 473 nm laser pulses and 1 mW for 530 nm constant laser, measured by an optical power meter (Thorlabs).

### 2.7. Neurological Behavioral Test and Brain Infarct Assessment

Modified neurological severity score (NSS) scaled from 0 to 14 [21] was used for the estimation of injury caused by ischemic insult and the recovery after NSC transplantation and laser stimulation. In addition, beam walk test was used to assess the neurological functional recovery. Before tMCAO surgery, animals were trained twice daily for 3 days to pass a beam with a length of 1 meter to establish the baseline. The assessment of NSS and beam walk test were performed at 3 and 14 days after tMCAO. Data were collected for three independent trials, and the average time was used in statistical analysis.

Animals were sacrificed at 14 days after tMCAO. Mice were transcardially perfused first with normal saline and then with freshly prepared 4% paraformaldehyde in PBS after deep anesthesia with 10% chloral hydrate (350 mg/kg). Brains were quickly removed into isopentane at −80°C and then frozen sectioned into 20 *μ*m slices. For each brain, five coronal sections that are 2 mm apart were used for the quantification of infarct volume. The sections were stained with 0.1% cresyl violet (Sigma) for 10 minutes at room temperature followed by gentle rinsing with water for 1 hour. The sections after staining were imaged using a digital camera, and the infarct volume was calculated using NIH ImageJ software as previously described [22]. The volume was assessed as the following formula:

(1) $$\begin{array}{c} V=\sum \frac{h}{3}\left[\Delta Sn+{\left(\Delta Sn*\Delta Sn+1\right)}^{1/2}+\Delta Sn+1\right]. \end{array}$$

In the formula, *h* represented the distance between two adjacent sections and Sn and Sn + 1 were the infarct areas of two adjacent sections. The infarct ratio equaled to the infarct volume divided by the ipsilateral brain volume in the same coronal plane.

### 2.8. Fluorescent Immunohistochemical Staining and Quantification

Brain sections or cells grown on glass coverslips were treated with 4% paraformaldehyde for 10 minutes, 0.3% Triton-PBS for 30 minutes, and 5% normal donkey serum for 60 min, followed by incubation with antibody at 4°C overnight. For NSC characterization after cell culture, Nestin and SOX2 antibodies were used for double-staining, both in a dilution of 1 : 200. For vascular density measurement, CD31 antibody (R&D Systems, Minneapolis, MN) was used in a dilution of 1 : 200. After thorough rinsing with PBS, samples were incubated with secondary antibodies (Invitrogen, Carlsbad, CA) in a dilution of 1 : 500 for 60 min and DAPI (Invitrogen) for 5 minutes at room temperature. Photomicrographs were taken with a confocal microscope (Leica, Solms, Germany) under a 40x objective lens. The fluorescent signal was computed by the software ImageJ. After binary conversion of the photomicrographs, the numbers of pixel which had a brightness exceeding threshold value were counted to calculate the proportion of fluorescent signal on each photomicrograph. Data were normalized to the control group. To measure the survival and migration of NSCs, images of the area around the needle passage of three brain slices per animal were collected and quantified to yield the average fluorescent area. In the assessment of local vascular density, three fields around the peri-infarct area were chosen from each brain slice, and four brain slices from each mouse were quantified to yield the average vascular density for each animal.

### 2.9. Apoptosis Assessment

Cell apoptosis in the peri-infarct area, where NSC was transplanted, was detected by TUNEL-DAB method using the ApopTag Peroxidase In Situ Apoptosis Detection Kit (Millipore, Bedford, MA). After TUNEL-DAB staining, the sections were counterstained with hematoxylin (Biyuntian Company, Shanghai, China). The areas around the injection track in the striatum were imaged under a 20x objective lens. The DAB-positive cells in each photomicrograph were counted and averaged over 6 brain sections for each animal.

### 2.10. Primary Neuronal Culture

Primary neuronal culture was prepared from the cortices of GAD2-ChR2-tdTomato transgenic mice at embryonic day 15. The cortices were quickly isolated in ice-cold calcium and magnesium-free Hanks balanced salts solution (HBSS) followed by digestion with 0.05% trypsin (Gibco) for 10 minutes. After three PBS rinses, cells were counted and plated onto poly-d-lysine-coated 6-well plates at 3 × 10^6^ per well in neural basal medium (Gibco) supplemented with 2% B27 (Gibco), 0.3 mM l-glutamine (Sigma), and 1% penicillin streptomycin. At 4 days after cell plating, 15 *μ*g/ml 5-fluoro-2′-deoxyuridine (Sigma) and 35 *μ*g/ml uridine (Sigma) were added to the cultures to inhibit nonneuronal cell proliferation. Half of the medium was replaced by fresh culture medium every 4 days. Neurons used for the experiment of laser stimulation were at 8 to 10 days of culturing.

### 2.11. Conditioned Medium Generation and NSC Viability Assessment

Before optogenetically stimulating the neurons, the medium was temporarily replaced by that used for NSC culture. The stimulation was carried out using 473 nm laser at 20 Hz pulses with a 5 ms pulse width and 0.05 mW power, for 5 minutes. The medium was immediately collected after stimulation. Standard cultured NSC spheres were dispersed into single cells using accutase and plated on a new well and incubated with the conditioned medium from excited GABAergic neurons for 2 days. The culturing medium was replaced by freshly conditioned medium daily.

The viability of the NSC in vitro was determined by CCK-8 kit (Donjindo, Kumamoto, Japan). The NSCs, cultured in conditioned media from light stimulated or nonstimulated GABAergic neurons, were plated in 96-well plates at 1 × 10^6^ cells/ml. Ten microliter of CCK-8 solution was added to each well and incubated for 2 or 12 hours at 37°C. The absorption data were acquired using a microplate reader (Synergy2 BioTek, Winooski, VT) at 450 nm.

### 2.12. Real-Time Polymerase Chain Reaction (PCR) Analysis

The total RNA from NSC or neuron was prepared by RNA-TRIZOL extraction (Gibco, Grand Island, NY). RNA concentration was determined by spectrometric methods (ND-3300, NanoDrop Technologies, USA). Reverse transcriptions of RNA to cDNA were performed via a universal cDNA synthesis kit (EXIQON, Vedbaek, Denmark). The amplification was performed by a fast real-time PCR system (7900 HT, ABI, Foster City, CA) using a SYBR Green master mix (EXQION). Primer sequences of the *β*-actin, NGF, and GDNF were obtained from the PrimerBank. The cycling conditions were 95°C for 10 minutes followed by 40 cycles of 95°C for 10 seconds and 60°C for 1 minute. The relative expression level of NGF and GDNF was normalized to the *β*-actin control in triplicate and was calculated using the 2-*Δ*ct method.

### 2.13. Statistical Analysis

All results were presented as mean ± SD. Data was analyzed by two-way (for behavioral tests only) or one-way (for other analysis) ANOVA followed by Bonferroni post hoc comparison using GraphPad Prism version 3.05 (GraphPad Software, Inc., La Jolla, CA). *p* < 0.05 was considered statistically significant.

## 3. Results

### 3.1. Validation of Optogenetic Control of Neuronal Activity in the Striatum

CaMKII promoter had been proved to be able to drive gene expression in most neurons in the striatum [20]. Consistent to the result of the previous study, in this study, AAV-carrying opsin gene under the control of CaMKII promoter successfully transfected most striatal neurons at 14 days after virus injection (Figure 1(b)).

**Figure 2:**
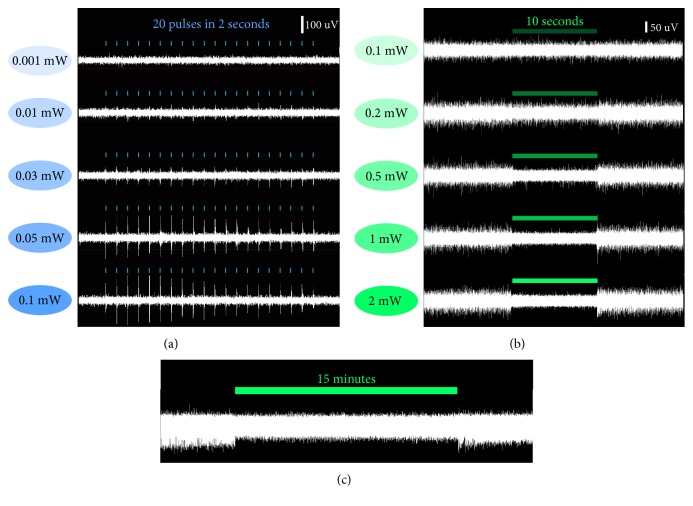
The validation of laser stimulation efficiency and the optimization of laser power based on electrophysiological recording in mouse striatum at 21 days after virus injection. (a) Excitatory stimulation with 473 nm laser pulse in the striatal area expressing ChR2. (b) Inhibitory stimulation with 530 nm constant laser in striatal area expression ArchT. (c) Uninterrupted stimulation with 1 mW 530 nm laser for 15 minutes did not affect the local spontaneous action potentials after the stimulation is finished.

In vivo electrophysiology data showed that the efficiency of optogenetic excitation or inhibition depended on the power of laser. A 0.05 mW 473 nm laser pulse was sufficient to stably trigger the action potential in striatal neurons expressing ChR2 (Figure 2(a)). For the striatal neurons expressing ArchT, administration of 530 nm constant laser with a power greater than 1 mW eliminated the majority of spontaneous action potentials (Figure 2(b)). The spontaneous action potentials immediately reappeared upon the conclusion of 530 nm laser stimulation (Figure 2(c)). These results demonstrated the successful optical control of striatal neuronal activity in this study.

### 3.2. The Inhibition of Striatal Neuronal Activity after Transplanting NSC Further Reduced Brain Infarct Volume after tMCAO

**Figure 3:**
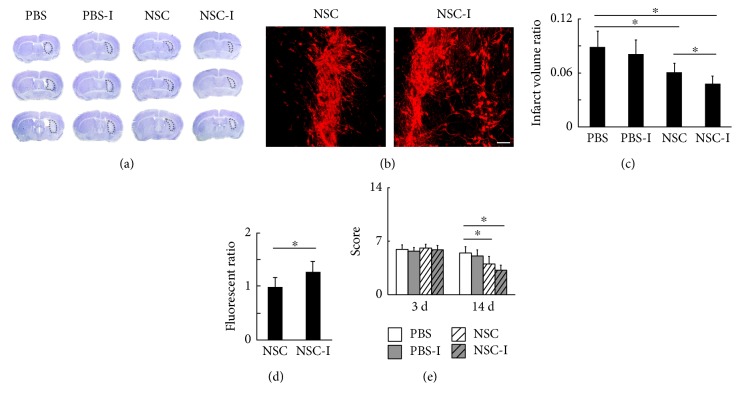
The inhibition of striatal neuronal activity enhanced the survival and migration of transplanted NSC and further reduced brain infarct volume. (a) Cresyl violet staining of the brain infarction at 14 days after tMCAO. (b) The fluorescence of NSC-RFP in the striatum at 14 days after tMCAO, bar = 100 *μ*m. (c) The ratio of infarct volume over the contralateral hemisphere volume (PBS = 8.6 ± 1.4%, PBS-I = 8.1 ± 1.6%, NSC = 6.3 ± 0.8%, and NSC-I = 4.5 ± 0.7%, *n* = 7). (d) The quantification of fluorescence signal from the transplanted NSCs (NSC = 1.00 ± 0.17, NSC-I = 1.29 ± 0.18, *n* = 6). (e) The behavioral outcome evaluated by NSS method at 3 days (PBS = 5.9 ± 0.6, PBS-I = 5.7 ± 0.4, NSC = 6.1 ± 0.4, and NSC-I = 5.9 ± 0.5, *n* = 7) and 14 days (PBS = 5.5 ± 0.9, PBS-I = 5.1 ± 0.8, NSC = 4.0 ± 0.9, and NSC-I = 3.3 ± 1.0, *n* = 7) after tMCAO. ∗ represents *p* < 0.05.

**Figure 4:**
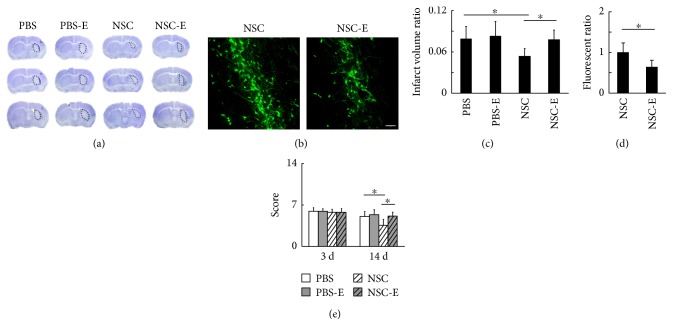
The excitation of striatal neurons after NSC transplantation reversed the beneficial effects of NSC in reducing brain infarction. (a) The brain infarction at 14 days after tMCAO. (b) The fluorescence from NSC-GFP in the striatum at 14 days after tMCAO, bar = 100 *μ*m. (c) The ratio of infarct volume over the contralateral hemisphere volume (PBS = 7.9 ± 1.8%, PBS-E = 8.3 ± 2.1%, NSC = 5.4 ± 1.1%, and NSC-E = 7.8 ± 1.4%, *n* = 7). (d) The quantification of fluorescence signal from the transplanted NSCs (NSC = 1.00 ± 0.23, NSC-E = 0.64 ± 0.16, *n* = 6) (e) The behavioral outcome evaluated by NSS method at 3 days (PBS = 5.7 ± 0.6, PBS-E = 5.7 ± 0.4, NSC = 5.6 ± 0.5, and NSC-E = 5.6 ± 0.6, *n* = 7) and 14 days (PBS = 4.8 ± 0.8, PBS-E = 5.1 ± 0.8, NSC = 3.4 ± 1.0, and NSC-E = 4.9 ± 0.7, *n* = 11) after tMCAO. ∗ represents *p* < 0.05.

The ratio of brain infarct volume in each group at 14 days after tMCAO was measured by cresyl violet staining (Figures 3(a), 3(c), 4(a), and 4(c)) followed by image quantification. In the set of inhibition experiments, the NSC group and NSC-I group showed significantly smaller ratio of brain infarct volume when compared to the PBS control (NSC = 6.3 ± 0.8% versus PBS = 8.6 ± 1.4%, *p* < 0.01; NSC-I = 4.5 ± 0.7% versus PBS = 8.6 ± 1.4%, *p* < 0.001, *n* = 7). The NSC-I group showed significantly smaller brain infarct volume when compared to the NSC group (NSC-I = 4.5 ± 0.7% versus NSC = 6.3 ± 0.8%, *p* < 0.05, *n* = 7), but the PBS-I group showed no statistical difference with the PBS group (PBS-I = 8.1 ± 1.6% versus PBS = 8.6 ± 1.4%, *p* > 0.05, *n* = 7). In the set of excitation experiments, the NSC group showed significantly smaller ratio of brain infarct volume when compared to the PBS control, PBS-E group, and NSC-E group (NSC = 5.4 ± 1.1% versus PBS = 7.9 ± 1.8%, *p* < 0.05; NSC = 5.4 ± 1.1% versus PBS-E = 8.3 ± 2.1%, *p* < 0.01; and NSC = 5.4 ± 1.1% versus NSC-E = 7.8 ± 1.4%, *p* < 0.05, *n* = 7). The NSC-E group showed no statistical difference with the PBS group. These results suggested that inhibition of striatal neuronal activity can further enhance the beneficial effect of transplanted NSC, while excitation of striatal neuronal activity abolished the beneficial effect of NSC in reducing brain infarct.

### 3.3. Excitation of Striatal Neuronal Activity Reversed the Beneficial Effect of Transplanted NSC in Promoting Neurological Functional Recovery after tMCAO

Neurological function was assessed using neurological severity score as previously reported at 3 and 14 days after tMCAO (Figures 3(e) and 4(e)). At 3 days after tMCAO, there were no statistical differences between any two groups. At 14 days after tMCAO, in the set of inhibition experiments, the NSC group and NSC-I group showed significantly lower NSS score when compared to the PBS control (NSC = 4.0 ± 0.9 versus PBS = 5.5 ± 0.9, *p* < 0.01; NSC-I = 3.3 ± 1.0 versus PBS = 5.5 ± 0.9, *p* < 0.001, *n* = 7). The NSC-I group showed no statistical difference to the NSC group. In the set of excitation experiments, the NSC group showed significantly lower severity score when compared to the PBS control, PBS-E group, and NSC-E group (NSC = 3.4 ± 1.0 versus PBS = 4.8 ± 0.8, *p* < 0.01; NSC = 3.4 ± 1.0 versus PBS-E = 5.1 ± 0.8, *p* < 0.01; and NSC=3.4 ± 1.0 versus NSC-E = 4.9 ± 0.7, *p* < 0.01, *n* = 11). The NSC-E group showed no statistical difference with the PBS group. The data of the beam walk test at 14 days after tMCAO showed that the NSC-I group is 15.1% faster in average to pass the beam when compared to the NSC group (NSC-I = 4.8 ± 0.9, NSC = 5.9 ± 1.4), but the NSC-E group took 20.1% more time in average to accomplish the mission when compared to the NSC group (NSC-E = 7.2 ± 1.7, NSC = 5.9 ± 1.4) (Supplementary Figure 1 available online at https://doi.org/10.1155/2017/4364302). These results suggested that excitation of striatal neuronal activity reversed the beneficial effect of transplanted NSC in promoting neurological functional recovery after tMCAO.

### 3.4. The Inhibition of Striatal Neuronal Activity Reduced the Apoptosis and Enhanced the Migration of Transplanted NSC

**Figure 5:**
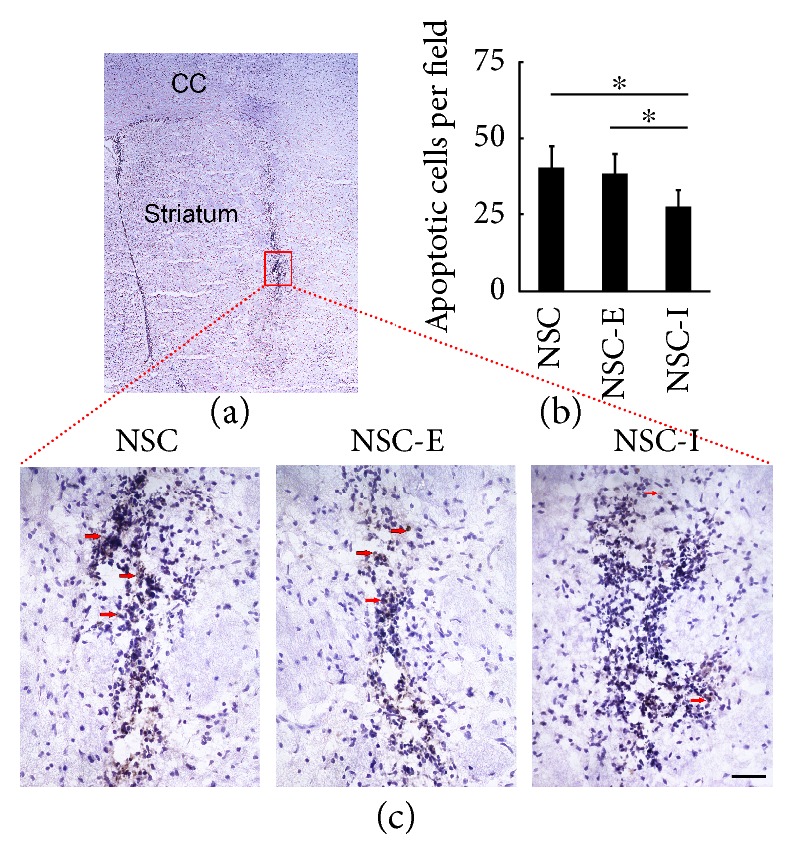
Striatal neuronal activity influenced cell apoptosis around the transplantation site. (a) DAB-TUNEL and hematoxylin staining. The area with high cell density in striatum is NSC transplantation site. The red rectangle shows the fields selected for quantifying the apoptotic cell number. (b) The quantitative result of apoptotic cell number in each group (NSC = 40.5 ± 6.6, NSC-E = 38.5 ± 6.2, and NSC-I = 27.7 ± 5.1, *n* = 6). (c) The apoptotic cells in NSC transplantation site. The brown signals indicated by red arrow are apoptotic cells. Bar = 20 *μ*m. ∗ represents *p* < 0.01.

Transplanted NSCs were derived from GFP or RFP transgenic mice and were characterized by staining for markers Nestin and SOX2 before transplantation (Figure 1(e)). The migration of the transplanted NSC was compared between the laser-stimulated group and nonstimulated group by determining the spreading of fluorescence-positive cells in the brain sections (Figures 3(b), 3(d), 4(b), and 4(d)). In the set of inhibition experiments, the NSC-I group showed significantly larger fluorescent area when compared to the NSC group (NSC-I = 1.29 ± 0.18 versus NSC = 1.00 ± 0.17, *p* < 0.05, *n* = 6). In contrast, in the set of excitation experiments, the NSC-E group showed significantly smaller fluorescent area when compared to the NSC group (NSC-E = 0.64 ± 0.16 versus NSC = 1.00 ± 0.23, *p* < 0.05, *n* = 6). The number of apoptotic cells around the needle path was compared between the laser-stimulated group and nonstimulated group after TUNEL-DAB staining (Figure 5()). The NSC-I group showed significantly less TUNEL-positive cells per field than the NSC-E group and NSC group (NSC-I = 27.7 ± 5.1 versus NSC-E = 38.5 ± 6.2, *p* < 0.01; NSC-I = 27.7 ± 5.1 versus NSC = 40.5 ± 6.6, *p* < 0.01, *n* = 6). These results suggested that inhibition but not excitation of striatal neuronal activity can reduce the apoptosis and enhance the migration of transplanted NSC.

### 3.5. The Inhibition of Striatal Neuronal Activity Further Increased the Vascular Density in the Penumbra after NSC Transplantation

**Figure 6:**
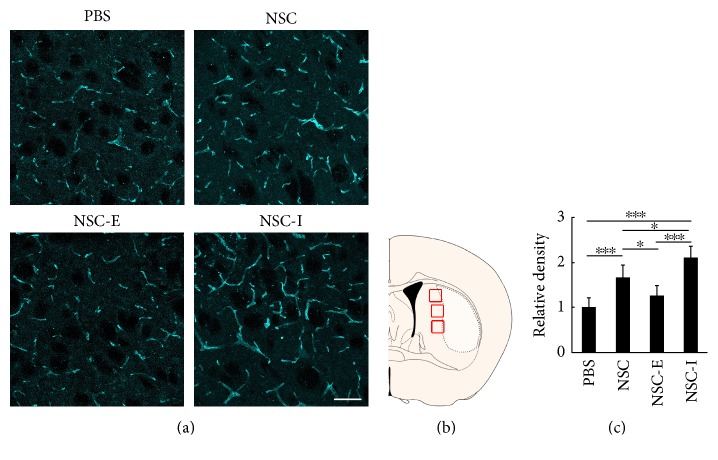
Striatal neuronal activity affected the vascular density in the penumbra after NSC transplantation. (a) The vascular density in the penumbra examined by staining with CD31, bar = 50 *μ*m. (b) Selected fields for imaging evaluation (indicated by red rectangles). The light-colored area represents brain infarct. (c) The quantitative result of vascular density (PBS = 1.00 ± 0.21, NSC = 1.67 ± 0.28, NSC-E = 1.27 ± 0.22, and NSC-I = 2.10 ± 0.26, *n* = 6). ∗ represents *p* < 0.05. ∗∗∗ represents *p* < 0.001.

To examine whether the improved survival and migration of transplanted NSC contributed to the better outcome after tMCAO through preventing vascular injury or promoting vascular repair, we examined the vascular density in brain sections by staining CD31 (Figure 6()). Three photomicrographs were taken in the penumbra area in each brain slice for quantification of the vascular density. The NSC group and NSC-I group showed significantly higher vascular density when compared to the PBS group (NSC = 1.67 ± 0.28 versus PBS = 1.00 ± 0.21, *p* < 0.001; NSC-I = 2.10 ± 0.26 versus PBS = 1.00 ± 0.21, *p* < 0.001, *n* = 6). In addition, the vascular density in the penumbra area of the NSC-I group was higher than that of the NSC group (NSC-I = 2.10 ± 0.26 versus NSC = 1.67 ± 0.28, *p* < 0.05, *n* = 6). In contrast, the NSC-E group exhibited reduced vascular density when compared to the NSC group (NSC-I = 2.10 ± 0.26 versus NSC = 1.67 ± 0.28, *p* < 0.05, *n* = 6). Compared to the PBS group, the NSC-E group showed a little higher vascular density in average, but the difference between the two groups is not significant (NSC-E = 1.27 ± 0.22 versus PBS = 1.00 ± 0.21, *p* > 0.05, *n* = 6). This result revealed a correlation between the enhanced transplanted NSC survival/migration and the increased vascular density in the NSC-I group and suggested that the better outcome after tMCAO brought by inhibiting the striatal neuronal activity after NSC transplantation is associated with higher vascular density.

### 3.6. Excitation of GABAergic Neuronal Activity Reduced NSC Viability through Paracrine Mechanisms in Cell Culture

**Figure 7:**
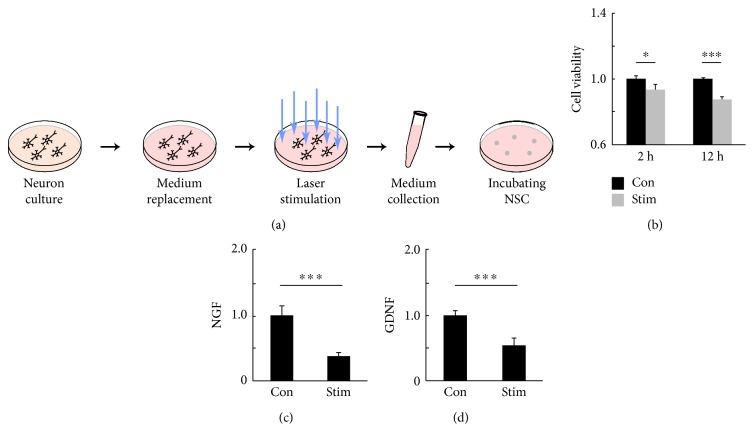
Excitation of GABAergic neurons can release factors that reduce NSC viability *in vitro*. (a) The schematic representation of the *in vitro* experiments. The blue arrows represent the stimulation with 473 nm laser pulse. (b) The viability of NSC, determined by CCK-8 assay at 2 hours (Con = 1.00 ± 0.03, Stim = 0.94 ± 0.02, *n* = 3) and 12 hours (Con = 1.00 ± 0.01, Stim = 0.88 ± 0.02, *n* = 3) after incubating with conditioned medium. (c) The relative mRNA expressions of NGF (Con = 1.00 ± 0.15, Stim = 0.37 ± 0.06, *n* = 3). (d) The relative mRNA expressions of GDNF (Con = 1.00 ± 0.07, Stim = 0.54 ± 0.10, *n* = 3). ∗ represents *p* < 0.05. ∗∗∗ represents *p* < 0.001.

To investigate whether striatal neuronal activity could influence the survival of transplanted NSC via paracrine mechanisms, we cultured neurons from GAD2-ChR2-tdTomato transgenic mice, stimulated the neurons by 473 nm laser pulse, collected the conditioned medium, and used the medium to incubate cultured NSC *in vitro* (Figure 7()). The control group used medium from unstimulated neuron for NSC culture. The viability of NSC was examined by CCK-8 method. The NSC treated by laser-activated-neuron-conditioned medium showed less viability at 2 hours (Stim = 0.94 ± 0.02 versus Con = 1.00 ± 0.03, *p* < 0.05, *n* = 3) and 12 hours (Stim = 0.88 ± 0.02 versus Con = 1.00 ± 0.01, *p* < 0.001, *n* = 3) after incubation when compared to the control (Stim = 0.94 ± 0.02 versus Con = 1.00 ± 0.03, *p* < 0.05, *n* = 3). To further determine the status of NSC, NSCs incubated by laser-activated-neuron-conditioned medium or unstimulated-neuron-conditioned medium were used for NGF and GDNF mRNA quantification. These two factors could contribute to the survival of NSC. The data showed that NSC treated by laser-activated-neuron-conditioned medium expressed significantly lower NGF (Stim = 0.37 ± 0.06 versus Con = 1.00 ± 0.15, *p* < 0.001, *n* = 3) and GDNF (Stim = 0.54 ± 0.10 versus Con = 1.00 ± 0.07, *p* < 0.01, *n* = 3) in mRNA level. These results indicated that excitation of GABAergic neuorns can induce the secretion of substances, which inhibit NSC viability in vitro, and suggested that striatal neuronal activity could influence the survival of transplanted NSC through paracrine mechanisms.

## 4. Discussion

It has been reported that transplantation of NSCs, alone or in combination with other progenitor/stem cells, could improve the functional recovery after ischemic stroke [9, 23, 24]. However, how the survival, proliferation, migration, and differentiation of implanted NSC are influenced by endogenous neuronal activity remains unclear. In this study, we used optogenetic technique to investigate the relationship between striatal neuronal activity and the survival of transplanted NSCs after ischemic stroke. Although CaMKII was traditionally considered as a specific promoter for excitatory neuron in the cortex, it had been proven that CaMKII could be used as a forebrain-neuron-specific promoter in striatum to drive the gene expression of inhibitory neurons [25]. The results from this work demonstrated that inhibiting striatal neuronal activities improved the survival and migration of transplanted NSCs and further reduced the brain infarct volume at 14 days after transient ischemic stroke. In contrast, activating striatal neuronal activities reversed the beneficial effect of transplanted NSCs in reducing brain infarct volume and promoting functional recovery. To investigate how neuronal activities affect the viability of transplanted NSCs, we cultured primary neurons from GAD2-ChR2-tdTomato transgenic mice and treated NSCs with the laser-activated-neuron-conditioned or unstimulated-neuron-conditioned medium (CM). The results showed that the CM from activated neurons decreased the expression of NGF and GDNF in NSCs. Reports showed that both NGF and GDNF play important roles in supporting of subpopulations of neurons [26–29]. This could be a potential reason why activating striatal neurons could decrease the survival of transplanted NSCs. The primary neurons used in the *in vitro* experiments of this study were cultured from the cortex consisted of approximately 20% GABAergic neurons [30]. The opsin-specific stimulation by laser would therefore activate about 20% of neurons in the culture. Nonetheless, we observed differences between the laser-excited group and the control group. A much significant difference may be observed with pure GABAergic neuron culture.

Studies have shown that exogenous stem cells could promote the functional recovery after ischemic stroke and other neurological diseases [24, 31–34]. However, one of the indisputable facts is that most of the transplanted exogenous stem cell would die in a short time after cell transplantation [16]. Our study demonstrated that inhibiting striatal neurons could promote the survival of transplanted NSCs after ischemia, providing a new sight to further augment the beneficial effect of NSCs.

## 5. Conclusions

NSC transplantation improved the neurological recovery after ischemic brain injury. Intrinsic neuronal activity impacts the migration and survival of transplanted NSCs. Optogenetic inhibition of striatal neuronal activity at the subacute phase after brain ischemia further augmented the beneficial effects of NSC transplantation, while activation of striatal neurons abolished the benefit of NSC treatment.

## Supplementary Material

Supplementary Figure 1. The time mouse spent to pass the beam in the beam walk test. No significant difference was observed in the four groups at 3 days before tMCAO (PBS ⁼ 2.9 ± 0. 7, NSC ⁼ 2.9 ± 0. 3, NSC-E ⁼ 2.8 ± 0.2, NSC-I ⁼ 3.0 ± 0. 4, *n*⁼6) and 3 days after tMCAO (PBS ⁼ 9.6 ± 2.3, NSC ⁼ 10.4 ± 2.1, NSC-E ⁼ 9.2 ± 1.9, NSC-I ⁼ 9.8 ± 1.7, *n*⁼6). At 14 days, NSC-I group passed the beam significantly faster than the PBS control group (NSC-I ⁼ 4.8 ± 0. 9 vs PBS ⁼ 7.7 ± 1.8, *n*⁼6). Though not significant, NSC-Igroupspend15.1% less time than NSC group (NSC-I ⁼ 4.8 ± 0. 9, NSC ⁼ 5.9 ± 1.4, *n*⁼6), NSC-E group spend 20.1% more time than NSC group (NSC-E ⁼ 7.2 ± 1.7, NSC ⁼ 5.9 ± 1.4, *n*⁼6). ^∗^ represents *p* < 0.05.
