# Supplementary material for: Optogenetic Inhibition of Striatal Neuronal Activity Improves the Survival of Transplanted Neural Stem Cells and Neurological Outcomes after Ischemic Stroke in Mice
Source: Stem Cells Int. 2017 Sep 14;2017:4364302. doi: 10.1155/2017/4364302 (PMC5618753; doi:10.1155/2017/4364302)
Supplement: Supplementary file 1 — Supplementary Figure 1. The time mouse spent to pass the beam in the beam walk test. No significant difference was observed in the four groups at 3 days before tMCAO (PBS ⁼ 2.9 ± 0. 7, NSC ⁼ 2.9 ± 0. 3, NSC-E ⁼ 2.8 ± 0.2, NSC-I ⁼ 3.0 ± 0. 4, n⁼6) and 3 days after tMCAO (PBS ⁼ 9.6 ± 2.3, NSC ⁼ 10.4 ± 2.1, NSC-E ⁼ 9.2 ± 1.9, NSC-I ⁼ 9.8 ± 1.7, n⁼6). At 14 days, NSC-I group passed the beam significantly faster than the PBS control group (NSC-I ⁼ 4.8 ± 0. 9 vs PBS ⁼ 7.7 ± 1.8, n⁼6). Though not significant, NSC-Igroupspend15.1% less time than NSC group (NSC-I ⁼ 4.8 ± 0. 9, NSC ⁼ 5.9 ± 1.4, n⁼6), NSC-E group spend 20.1% more time than NSC group (NSC-E ⁼ 7.2 ± 1.7, NSC ⁼ 5.9 ± 1.4, n⁼6). ∗ represents p < 0.05. [file 4364302.f1.docx]

Supplementary Figure1
